# Data exploration, quality control and testing in single-cell qPCR-based gene expression experiments

**DOI:** 10.1093/bioinformatics/bts714

**Published:** 2012-12-24

**Authors:** Andrew McDavid, Greg Finak, Pratip K. Chattopadyay, Maria Dominguez, Laurie Lamoreaux, Steven S. Ma, Mario Roederer, Raphael Gottardo

**Affiliations:** ^1^Department of Statistics, University of Washington, Seattle, WA 98195, USA, ^2^Vaccine and Infectious Disease Division, Fred Hutchinson Cancer Research Center, Seattle, WA 98109, USA, ^3^ImmunoTechnology Section, Vaccine Research Center, NIAID, NIH, Bethesda, MD 20892, USA and ^4^Immunology Laboratory, Vaccine Research Center, NIAID, NIH, Bethesda, MD 20892, USA

## Abstract

**Motivation:** Cell populations are never truly homogeneous; individual cells exist in biochemical states that define functional differences between them. New technology based on microfluidic arrays combined with multiplexed quantitative polymerase chain reactions now enables high-throughput single-cell gene expression measurement, allowing assessment of cellular heterogeneity. However, few analytic tools have been developed specifically for the statistical and analytical challenges of single-cell quantitative polymerase chain reactions data.

**Results:** We present a statistical framework for the exploration, quality control and analysis of single-cell gene expression data from microfluidic arrays. We assess accuracy and within-sample heterogeneity of single-cell expression and develop quality control criteria to filter unreliable cell measurements. We propose a statistical model accounting for the fact that genes at the single-cell level can be on (and a continuous expression measure is recorded) or dichotomously off (and the recorded expression is zero). Based on this model, we derive a combined likelihood ratio test for differential expression that incorporates both the discrete and continuous components. Using an experiment that examines treatment-specific changes in expression, we show that this combined test is more powerful than either the continuous or dichotomous component in isolation, or a *t*-test on the zero-inflated data. Although developed for measurements from a specific platform (Fluidigm), these tools are generalizable to other multi-parametric measures over large numbers of events.

**Availability:** All results presented here were obtained using the SingleCellAssay R package available on GitHub (http://github.com/RGLab/SingleCellAssay).

**Contact:** rgottard@fhcrc.org

**Supplementary information:** Supplementary data are available at *Bioinformatics* online.

## 1 INTRODUCTION

The development of fluorescence-based flow cytometry (FCM) revolutionized single-cell analysis. Although populations of cells sorted by FCM using surface markers may appear monolithic, mRNA expression of specific genes within these cells can be heterogeneous (Dalerba *et al.*, 2011) and could further discriminate cell subsets. On the other hand, classical gene expression experiments [microarrays, RNA-seq, quantitative polymerase chain reactions (qPCR)] richly characterize a cellular population but at the cost of reporting a summation of expression from many individual cells. Recent advances in microfluidic technology now permit performing thousands of PCRs in a single device, enabling gene expression measurements at the single-cell level across hundreds of cells and genes (Kalisky and Quake, 2011). This provides a technology that probes the stochastic nature of biochemical processes, resulting in relatively large cell-to-cell expression variability.

This heterogeneity may carry important information; thus, single-cell expression data should not be analysed in the same fashion as population-level data. At the scale of a single cell, biological variability (the object of interest) and technical variability (a nuisance factor) are often of the same magnitude, making it difficult to distinguish between the two. No expression (i.e. the gene is off) may be detected in individual cells owing to real biological effects, resulting in zero-inflation of otherwise continuous measures. These features of single-cell data require special attention during analysis.

Here, we focus on the reverse-transcriptase qPCR (rt-qPCR)-based Fluidigm (San Francisco, CA) single-cell gene expression assay, which provides simultaneous measurements of up to 96 genes on mRNA sources as minute as a single cell. In traditional rt-qPCR, despite careful measurement of input cDNA concentrations, differences in starting material below the limit of detection require correction for reliable results (Vandesompele *et al.*, 2002). Subtraction of internal control genes, or averages thereof is typically used (e.g. the 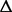-Ct method), and results are often reported in fold increase per cell (Schmittgen and Livak, 2008). In array-based gene expression, differences in hybridization and washing of non-specific DNA between chips require additional correction.

Such normalization schemes are not directly applicable in single-cell gene expression experiments, nor is it obvious that they are needed. For single cells, the individual cell is the atomic unit of normalization and the amount of starting material naturally measured in number of cells per reaction. Even if one attempted direct application of traditional normalization approaches, the dichotomous nature of single-cell expression hinders their use.

Nonetheless, it is important to test for and address any technical biases. We present a filtering approach for removing outlying measurements at the single-cell level that accounts for the dichotomous nature of the data. Using concordance measures derived from three datasets where gene expression was measured at the single-cell and 100-cell levels, we show that classical rt-qPCR type normalization is not necessary with single-cell multiplexed PCR data, and that our filtering step removes technical artifacts that most severely impact quantitation.

A typical goal of gene expression experiments is to search for differential expression across groups. The zero-inflation of expression in Fluidigm introduces problems for testing differential representation of cell subsets characterized by expression patterns, as well. Traditional tests of differential expression such as the *t*-test or other approaches based on normality are likely inappropriate for zero-inflated data (Gottardo *et al.*, 2006; Smyth, 2004). Approaches to this problem have varied. Powell *et al.* (2012) used a winsorized z-transformation of the expression values and then treated them as continuous. Glotzbach *et al.* (2011) used the non-parametric, Kolmorgov–Smirnov test for differences in distribution to find differentially expressed genes after winsorizing. Flatz *et al.* (2011) dichotomized the expression and worked with the binary trait. Of these authors, only Flatz *et al.* (2011) and Glotzbach *et al.* (2011) made use of formal tests of differential expression. However, as we will see later, both the continuous and discrete parts of the measurements are informative for differential expression and should be used. A parametric test allows directions of difference to be assessed.

Here, we propose a discrete/continuous model for single-cell expression data based on a mixture of a point mass at zero and a log-normal distribution. Using this model, we derive a likelihood ratio test (LRT) that can simultaneously test for changes in mean expression (conditional on the gene being expressed) and in the percentage of expressed cells.

## 2 METHODS

### 2.1 Datasets and notations

We use three Fluidigm single-cell gene expression datasets described later in the text. We offer a brief overview of the assay technology used for our data. Desired cells (e.g. antigen-specific CD8+ T cells) are selected and lysed, and a cDNA library is generated through rt-qPCR. A short (c. 15 cycle), multiplexed pre-amplification selects and enriches for the desired genes. These products are loaded onto the Fluidigm chip, and gene-specific primers are added for single-cell gene expression quantitation. For the data presented here, we used a 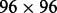 format plate, i.e. 96 genes across 96 cells. The design of the chip generates each combination of the 96 genes and 96 enriched cDNA libraries producing 9216 separate PCR reactions. After each cycle, the fluorescence is read. The cycle (or interpolated fraction thereof) at which the fluorescence crosses a pre-determined threshold is recorded, defined as the ‘*ct*’ value. For all datasets considered here, primers were chosen to have 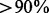 amplification efficiency.

**Dataset A:** Twenty-eight 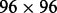 format plates of CMV- or HIV-specific CD8+ single cell T cells were isolated from 16 individuals. The donors’ cells were stimulated with one of four tetramers. Cells were sorted immediately after tetramer incubation (‘unstimulated’) or after 3 hours of exposure (‘stimulated’). Approximately 90 individual cells were measured for each patient-stimulation combination (‘unit’).

**Dataset B:** Ten subjects were considered, and ∼180 activated CD4+ memory T cells were sorted per subject, with each subject crossed between two arrays.

**Dataset C:** Two subjects were considered. Fluorescent staining of CD4+ T cells allowed cytometric sorting into CD154+/− sub-populations. Approximately 40 cells were sorted per sub-population per subject across three arrays.

Additionally, for each individual and treatment within each dataset, aggregates of 100 cells (i.e*.* 100 cells per well on the array) were isolated and assayed by Fluidigm technology. The expression measured in these 100-cell aggregates, after dividing by 100, provides a ‘biological’ average of expression per cell and can be compared with an *in silico* average of the single-cell measurements. The *concordance* between these two averages serves as a measure of experimental fidelity (Lin, 1989).

**Notations:** The standard assumptions of qPCR-based assays apply to the Fluidigm technology, namely that the cycle threshold (*ct*) is inversely proportional to the log of fluorescence. The fluorescence is directly proportional to the starting concentration of mRNA (Higuchi *et al.*, 1992; Karlen *et al.*, 2007). The Fluidigm instrument returns the cycle threshold (*ct*); however, we find it more useful to work with the complement of *ct*, which we define as the *expression threshold* (*et*)

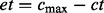

where 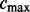 is the maximum number of cycles used, 40 in our case. Assuming all reactions are in the exponential amplification phase, this quantity should be directly proportional to the log-abundance of mRNA, plus an intercept term corresponding to the number of cycles it takes for the minimally detectable quantity of mRNA to cross threshold. If the fluorescence does not cross the threshold after 40 cycles, then the Fluidigm instrument records a value of N/A, and we say that the gene is *not detected*. As we will see in the Section 3, detected genes typically have a value of *ct* much less than 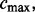 suggesting that undetected genes might be regarded as unexpressed genes. This assumption is supported by the idea that transcription of mRNA is thought to occur in bursts of activity (Kaufmann and van Oudenaarden, 2007; Levsky *et al.*, 2002), followed by quiescence. Other authors have noted this feature in single-cell expression studies as well (Glotzbach *et al.*, 2011). When looking at the concordance of the single-cell and 100-cell experiments, this assumption is reasonable and leads to better concordance than omitting the N/A values. As a consequence, we treat the undetected genes as unexpressed genes, and we set the corresponding *et* value to 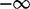 so that the mRNA abundance is zero (i.e. 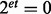).

For a fixed sample or experimental unit, let us denote by 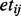 the expression threshold of *well* 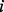 and *gene* 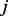, for 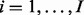 and 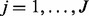. This results in a matrix of 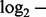
based expression values, 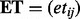, just as in array-based gene expression. Similarly, we will denote by 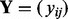 the matrix of untransformed expression values, where 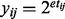. Usually, a well contains one cell, but the Fluidigm technology can be used with multiple cells per well to quantify the gene expression of a mixture of cells. As a consequence, we prefer to use the term ‘well’ instead of ‘cell’. In the three datasets used here, wells will contain either 1 or 100 cells. Finally, several biological units are typically measured in an experiment, and in this case, we will use an extra index 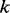 to refer to the biological units.

### 2.2 A model for single-cell expression

**Fig. 1.**
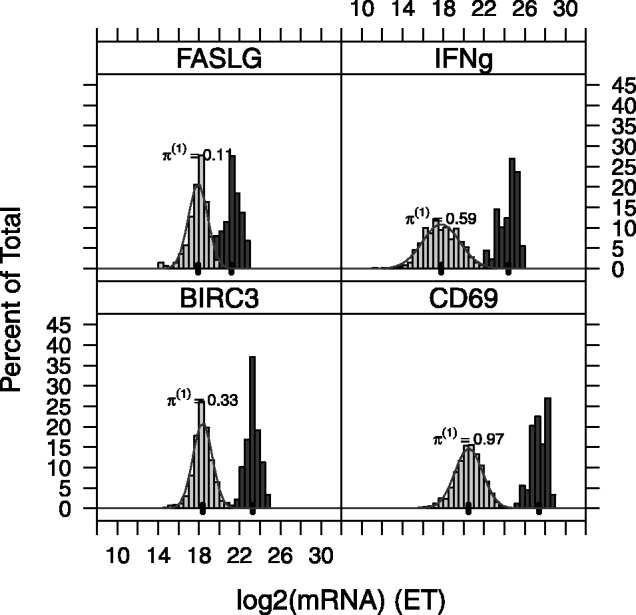
Histogram and theoretical (normal) distribution of 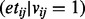 for single-cell (left, light gray) and 100-cell experiments (right, dark gray). Genes FASLG, IFN- 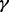, BIRC3 and CD69 are depicted. The frequency expression of each gene in the single-cell experiments 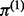 is printed above each histogram. The mean of the 100-cell and single-cell experiments is indicated by a thick black line along the *x*-axis

As described previously, for a given cell, a gene can be defined as *on* (i.e*.* a positive *et* value is recorded) or as *off* (i.e*.* the gene is undetected and 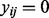). To simplify our model, we will denote by 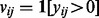 the indicator variable equal to one if the gene 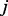 is expressed in well 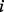 and zero otherwise. Following classical statistical conventions, we use upper cases to denote the random variables and lower cases to denote the values taken by these random variables. Using these notations, we introduce the following model of single-cell expression
(1)
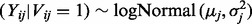

(2)
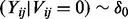

(3)
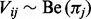

where 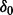 denotes a point mass at zero, 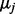 and 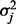 are the 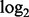-based mean and variance expression-level parameters conditional on the gene being expressed (i.e. 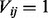), and 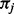 is the frequency of expression of gene 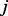 across all cells. In the datasets considered here, the frequency of expression greatly varies across genes from 0 to 0.99 with a median value of 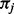 ∼0.1 (see Supplementary Fig. S1). Assuming a log-Normal model for 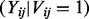 is equivalent to modeling 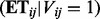 as normally distributed. The empirical distribution of the data (Fig. 1 and Supplementary Figs S8–S10) motivates our selection of a log-normal distribution and follows observations of previous authors (Bengtsson *et al.*, 2005).

Thus, in a particular gene, three parameters characterize the expression distribution: 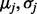, the mean and standard deviation of the 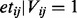, and 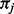, the Bernoulli probability of expression.

### 2.3 Quality control and filtering

The Fluidigm assay is sensitive, and owing to the exponential amplification of starting mRNA, even minute contamination can render a measurement unreliable. Similarly, variation in cell preparation can have significant impact on the resulting experiment and data, such as unintentional empty wells, which would distort estimates of 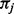. This suggests identifying, and possibly removing outliers before conducting further analysis. We examine both the discrete component 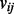 and the continuous component 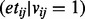 in screening for outliers. We define the robust z-transformed positive expression value as

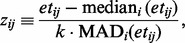

where the median and median absolute deviation (MAD) are calculated, for a given gene, over expressed cells (i.e. 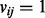), and 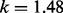 is a scaling constant that gives the standard deviation in terms of the MAD for the normal distribution. Next, let 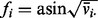 be the Bernoulli variance-stabilizing transformation of the proportion of genes expressed in well 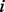. Then, we define the robust z-transformed fraction as

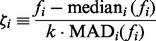

where the median, MAD and 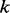 are as defined previously. This leads to the following steps for filtering:

1. Remove *null* cells with no detected genes, i.e. 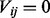, for all 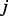.
2. Pick threshold for *z* filtering (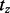); threshold for 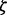 filtering (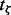).
3. Calculate 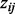 and 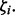
4. Remove wells in which genes have 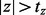 OR 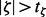.

Step 1 removes wells where no cells were loaded, and thus all measured expression values are null. It is important to perform this step first to prevent break-down in the median and MAD estimates for the zeta values in experiments with many amplification or FCM failures. Finally, step 4 removes unreliable wells that either have an extreme proportion of expression or extreme cell 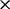 gene expression values. The thresholds 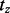 and 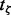 control the tolerance to outliers; therefore, typical advice for outlier thresholding applies. Biological replicates, such as the 100-cell replicates described in Section 2.1, permit the assessment of intra-class deviance, and then the thresholds can be selected to minimize this deviance. We present such a calculation in the Supplementary Material. Using this approach, we find that picking 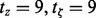 works well for the datasets we consider here, see Section 3.

### 2.4 Testing for ET differences between experimental groups

One typical goal of gene expression analysis is to test for difference in expression patterns between experimental units. Here, we focus on testing differential gene expression between two paired-biological units, e.g. before and after stimulation. Our framework should be generalizable to other types of situations, see Section 4. The classical test for changes in mean for samples with continuous measurements is the 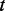-test. Conversely, if only a change in 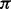 were of interest, then a contingency table test (Chi-square, Fisher’s exact or Bernoulli likelihood ratio) is appropriate. However, in our case, we would like to test for a change in 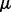 and 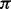 simultaneously, as both could be biologically relevant. Formally, we wish to test

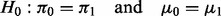

versus the alternative

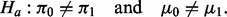



This can be accomplished using an LRT that would simultaneously test for differences in means or proportions of expression.

Suppose that 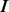 wells are assayed in each unit, though the unbalanced case (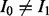) would be treated similarly with obvious changes of notation. Based on (1), the likelihood function for one gene across two biological units, omitting the gene index 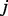 for clarity, is given by
(4)
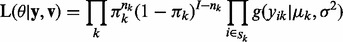

where 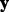 and 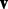 are the vectors of observations for the gene across the two groups, 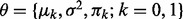 is the vector of unknown parameters, 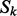 is the set of cells expressing the gene in group 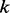 (i.e. 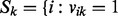}), 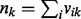 is the number of cells expressing the gene in group 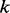 and 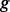 is the density function of the log-normal distribution with parameters 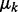 and 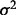. The LRT statistic 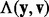 is then defined as the ratio of the null and alternative likelihoods obtained by replacing the unknown parameters with their null and alternative maximum likelihood estimates. Detailed derivations of the likelihood function and the LRT statistics are described in Supplementary Material.

An interesting observation is that the likelihood function given by (4) is the product of the Bernoulli likelihood for all cells and the log-normal likelihood for the expressed cells. It follows that the log-LRT statistic decomposes as a sum of a Bernoulli log-LRT test statistic and a log-normal log-LRT test statistic, as each component can be maximized independently. It thus combines the two sources of information in a natural way, and this decomposition allows post-hoc assessment of which of the component(s) drive the detected difference by simply comparing the magnitude of the two log-LRTs. In Section 3, we will show that our combined LRT test is more powerful than the Bernoulli or log-normal tests alone.

Applying classical asymptotic results about LRTs (Wilks, 1938), 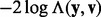 converges to a 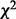 distribution with two degrees of freedom under 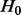. Some care is warranted in invoking this asymptotic result, as even for large 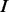, the sample size for the log-normal LRT will be 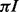. We show in Supplementary Figs S2 and S3 that the 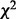 convergence is adequate for 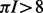 even under departures from normality. Below this value, it is possible to derive the null distribution of this statistic through permutation procedures as is commonly done for microarray data (Ge *et al.*, 2003). This proviso applies similarly for purpose of power calculations; hence, one may wish to conduct these through simulation.

## 3 RESULTS

### 3.1 Distributional assumptions

In Figure 1, we observe good agreement between the empirical distributions of positive *et* values and their postulated normal distribution for four genes in dataset A. This confirms that a log-normal model for the positive expression level, 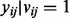, is appropriate. Even cells in the lowest quantiles of *et* (and lowest quantiles of expression) still have expression far away from the bound at 0, suggesting that undetected genes represent cells with null or negligible RNA abundance. It is also noteworthy that the difference between the means (shown as a heavy, vertical line) of the 100-cell replicates and single-cell replicates is approximately 
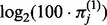
 cycles, where 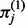 is the expression frequency of gene 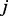 in the single-cell experiments. As such, in genes with 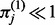, such as FASLG, this difference between means is smaller than genes with 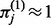. As we will see the next section, inclusion of the unexpressed cells (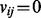) is important to accurately relate the expression level of the single-cell experiments to the 100-cell experiments.

### 3.2 Concordance between 100-cell and single-cell experiments

The 100-cell aggregates (see Section 2.1) allows us to assess the accuracy and reliability of our single-cell experiments by comparing this *in vitro* 100-cell expression to an *in silico* estimate obtained by averaging the expression of 100 single-cell measurements. The *in silico* average of signal in a gene 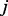 and unit 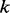 from 100 single-cell wells is 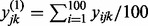 where 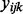 is the expression measurement of gene 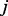 in cell 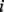 and unit 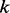. We compare this with the *in vitro* ‘average’ of signal from a 100-cell aggregate. In this case, we just use the expression of a gene unit and divide by the number of cells (100).

**Fig. 2.**
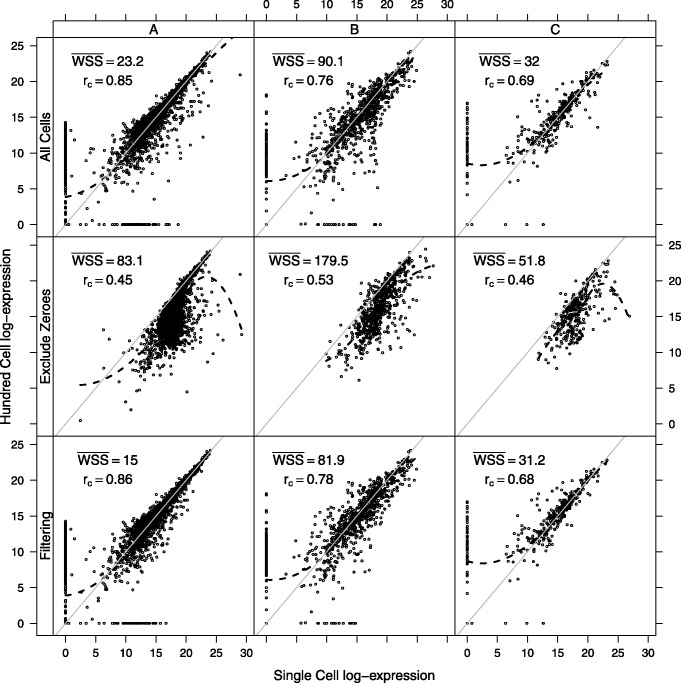
Concordance between 100 cell 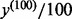 and 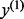, the *in silico* average of single-cell wells for datasets A, B and C. In the top row, wells with 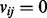 are included and treated as exact zeroes. In the middle row, they are excluded, resulting in a clear lack of concordance. In the final row, wells are filtered as per Section 2.3. Dark, thin lines show the initial location of a gene before filtering and connect to the location of the gene after filtering. In each panel, 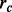, the concordance correlation coefficient and 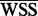, the average weighted squared deviation of expression measurements is printed. The dotted black line shows a loess fit through the data. In all cases, the expression values are transformed using a shifted log-transformation [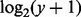]. As such, a graphed value of zero corresponds to a zero expression value (i.e. 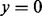)

The concordance here is assessed both visually by plotting 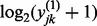 versus 
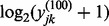
 (Fig. 2) and by calculating the concordance correlation coefficient (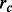) between the two variables, which is often used to quantify reproducibility (Lin, 1989). The shifted log transformation allows visualization of both the discrete and continuous components while being on the *et* scale.

We first use this concordance experiment to test whether wells that do not cross the fluorescence threshold after 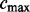 should be treated as exact zeroes or missing values. If we suppose that 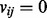 implies an assay failure and the measurement should be discarded, we would simply compute the single-cell average over expressed cells, i.e. 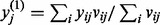. Figure 2 demonstrates good concordance between the 100-cell and single-cell experiments when the undetected genes are treated as zeros. However, this is not the case when the zeros are treated as missing values.

### 3.3 Filtering outlying cells

In addition to the concordance measure 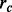, we use another goodness-of-fit measure to optimize our filtering parameters 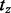, 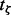 defined by,
(5)
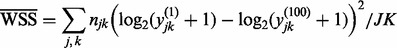

where 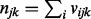 is the number of positive wells for gene 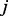 in unit 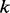 in the single-cell experiments. For a particular gene and unit, the 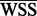 decreases as we lower the filtering threshold and extreme values are filtered. Eventually, so many cells are removed that there is zero expression (and a large deviance) for the *in silico* estimate. Thus, we wish to find a set of values for the filtering parameters that would lead to the lowest 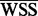 measure across the three datasets used here. The addition of the scaling factor 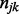 gives higher weight to combinations with more *ex ante* positive observations so that the contribution to the sum of squares would be smaller in gene 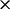 unit combinations that have fewer expressed cells. The factor 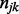 can also be interpreted as the scaling factor for the variance of the mean over positive observations. Finally, the 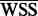 is computed on the 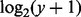 scale to reduce the effect of extreme outliers.

When 100-cell aggregates are available, one can optimize the filter parameters 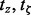 by minimizing the 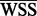 over possible combinations. In our case, we found that setting 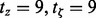 achieves the best reduction in 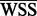 across the three datasets explored here (Supplementary Figs S4–S6 and Supplementary Table S1). Using these values, our filtering criteria moderately improve the concordance between the single-cell and 100-cell experiments in two of the datasets but dramatically improve (decrease) the weighted sum of squares. This improvement is evident graphically, as the per unit averages of *et* of multiple genes move toward the diagonal.

Beside improving 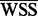 and generally improving 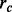, we explore the effect of filtering on detection of control genes in the Supplementary Material (Supplementary Table S2).

### 3.4 Normalization and housekeeping genes

Other authors have noted that ‘the gene transcript number is ideally standardized to the number of cells’ (Vandesompele *et al.*, 2002), which is the case with gene expression from sorted cells. Therefore, it is not entirely a surprise that we find little evidence for housekeeping genes providing useful normalization here. For a housekeeper to have good validity, it should have high cross-correlation with other housekeeping genes. This is not the case for housekeepers GAPDH and POLR2A, which in dataset A, in linear regression, have an 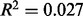. In Supplementary Figure S7, we observe in scatter plots of housekeepers’ *et* that the correlation drops even further (to an 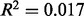) after filtering outlying cells (see previous section). As the correlation between housekeepers is present primarily in cells we suspect suffered from technical error, we find little utility in normalization schemes. In fact, the use of housekeeping genes for normalization could even result in masking cellular artifacts that should be filtered.

### 3.5 An efficient test of differential expression for single cells

**Fig. 3.**
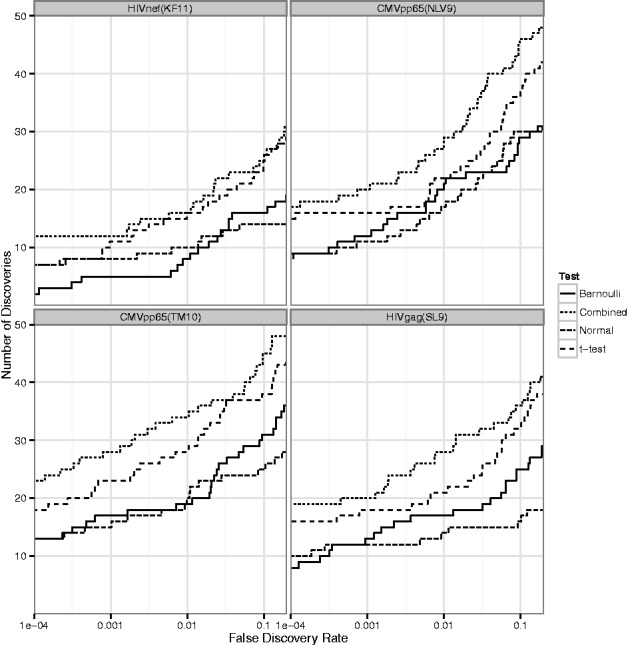
Number of discoveries (genes 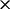 units) versus FDR, by treatment, dataset A. The combined LRT is compared with a Bernoulli or normal-theory only LRT, as well as a *t*-test of the raw expression values (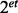 scale), including zero measurements

In dataset A, ∼90 cells in each of 16 subjects were measured in unstimulated and stimulated states (see Section 2.1). This permits conducting a test for each gene in each subject for differences in 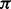 and 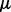, as described in Section 2.4. We plot the number of discoveries at various false discovery rates (FDR) in Figure 3. The combined likelihood test produces the greatest number of discoveries over a wide range of FDR. For example, at an FDR of 1%, our combined test could detect more than 20 additional gene 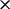 unit changes across the four stimulations.

**Fig. 4.**
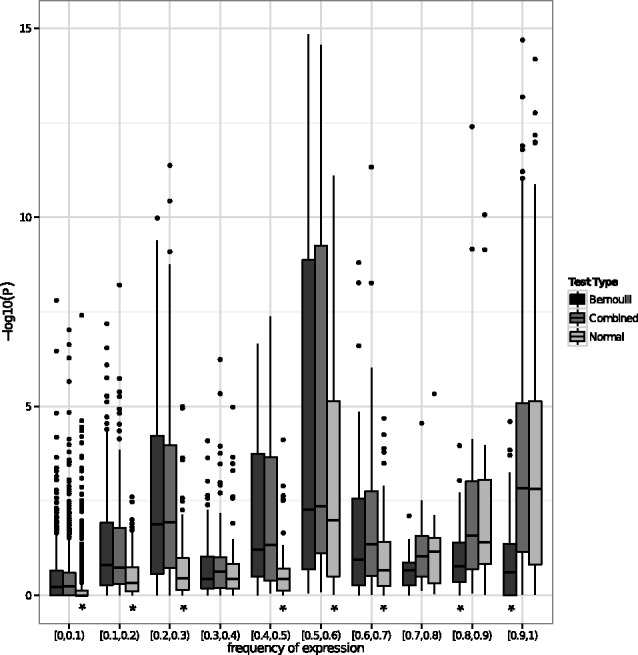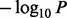 of tests (genes 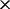 units) versus frequencies of expression 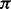 of the genes. The Bernoulli, normal-theory and combined LRTs are plotted. Asterisk indicates test is different from the combined test at 5% significance in a Wilcoxon signed-rank test

Another feature of the combined LRT is its robustness to background gene frequency 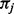. Of course, if 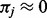 on average, then any test will be underpowered to detect group differences. But using only the continuous components amounts to “throwing away” data for genes with intermediate 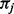. And similarly, using only the dichotomous component results in a test insensitive to differences in 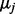 in frequently expressed genes. This robustness to the 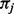 spectrum is shown in Figure 4 in which 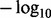 *P*-values are shown for the Bernoulli, normal and combined LRTs versus frequency of 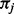.

**Fig. 5.**
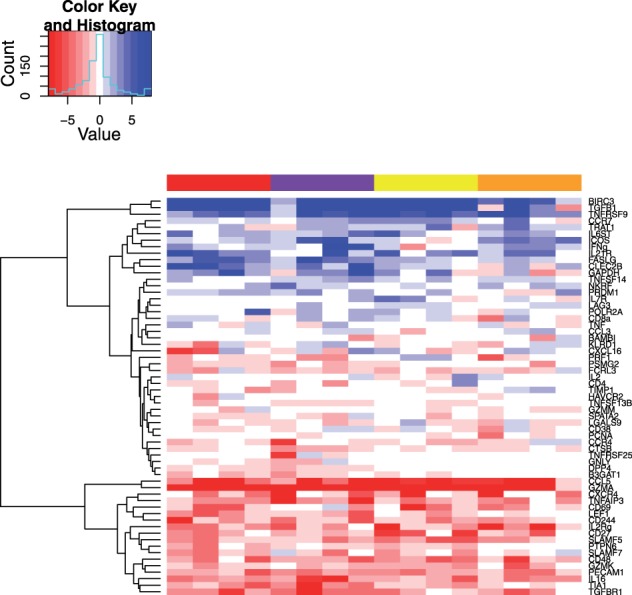
Heatmap of signed 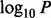 for selected genes (rows, see main text) and all 16 individuals (columns). The color above each column indicates the antigen stimulation applied to the cells; thus, individuals are randomly arranged in each antigen block. Red and purple are two different CMV antigen pools; yellow and orange are two different HIV antigen pools

A total of 65 genes were detected at an FDR of 1% in at least one individual. We define 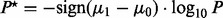 as the negative 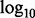 *P*-value times an indicator variable, which is positive when stimulated groups have greater expression, and negative otherwise. Figure 5 plots a heatmap of signed 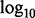 *P*-values. The selected genes are in clustered rows; the 16 individuals are arranged in columns by stimulation. The color above each column indicates which of the four antigen stimulations the individual received. From this, it is clear that genes cluster into upregulated and downregulated modules, and that there is much individual variability in response. Some genes appear to have stronger responses to particular antigens, such as the response to CMV (red and purple columns) in FASLG and CLEC2B.

## 4 CONCLUSIONS

Current approaches for analysis of single-cell assays have incompletely used the salient features of the experiment, and the resulting inference can be suboptimal. In this article, we have presented a framework for data exploration, quality control and testing for differential expression using single-cell data. Our comparison of 100-cell and single-cell measurements shows that undetected genes in an assay should be treated as effective ‘zeroes’. Both the discrete, zero-inflated portion and continuous portion of single-cell expression data are meaningful for detecting outliers. Moreover, differences in either could be of biological interest; therefore, it is desirable to combine evidence from both to detect changes in expression. Our LRT allows just that.

Although we have suggested default parameters for the filtering of outliers, informed from several datasets, our defaults are likely conservative. They are 3–4 times larger than the most substantial difference in expression between experimental groups we observed. Acquiring forms of ground-truth besides ‘bulk’ experiments (in our case, 100-cell aggregates) could allow forming tighter bounds. As in any outlier-based filtering procedure, it is desirable to tune for the problem at hand. The thresholds 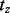 and 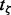 should be different when eliminating potential technical error is of greatest concern than when one is most interested in searching for biological heterogeneity.

In this article, we have used the 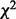 asymptotic distribution of the LRT to compute *P*-values and assess significance. This approximation is relatively accurate and robust to the distributional form of 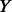 when the expected number of expressed cells is greater than 8 (see Supplementary Material). Otherwise, approximating the null distribution using permutations as in Ge *et al.*, 2003 is more appropriate.

Further work, incorporating a mixed-effects model to our LRT, could extend its applicability. The test outlined in this article may not be appropriate in cases where traits of interest are not blocked within individuals (e.g*.* comparing between phenotypes like HIV+ versus HIV−). In this case, one wishes to identify gene expression changes across groups, despite high individual-to-individual heterogeneity. By modeling the mean and proportion of expression as common across groups and adding specific random effects for between-individual variability, our model could be extended to address such experimental questions as well.

Single-cell gene expressions assays have already been shown to be useful in multiple studies and will become even more routine once sequencing at the single-cell level becomes practical (Ramskold *et al.*, 2012; Varadarajan *et al.*, 2011). As a consequence, the development of effective statistical methods to analyse such data is becoming increasingly important. This article offers a coherent framework for researchers using this nascent technology.

*Funding*: Intramural Research Program of the National Institute of Allergy and Infectious Diseases, National Institute of Health and the Collaboration for AIDS Vaccine Discovery [#38650]; National Institute of Health [U19 AI089986-01, R01 EB008400 to R.G., G.F. and A.M.]; and the Bill & Melinda Gates Foundation [#OPP1032325].

*Conflict of Interest*: none declared.

## Supplementary Material

Supplementary Data
