# Supplementary material for: Data exploration, quality control and testing in single-cell qPCR-based gene expression experiments
Source: Bioinformatics. 2012 Dec 24;29(4):461–7. doi: 10.1093/bioinformatics/bts714 (PMC3570210; doi:10.1093/bioinformatics/bts714)
Supplement: Supplementary Data [file supp_29_4_461__index.html]

Data exploration, quality control and testing in single-cell qPCR-based gene expression experiments — Data exploration, quality control and testing in single-cell qPCR-based gene expression experiments — Supplementary Data 

# Data exploration, quality control and testing in single-cell qPCR-based gene expression experiments

## Supplementary Data

files

**Files in this Data Supplement:**

- Supplementary Data - pdf file
