# Supplementary material for: Data exploration, quality control and testing in single-cell qPCR-based gene expression experiments
Source: Bioinformatics. 2012 Dec 24;29(4):461–7. doi: 10.1093/bioinformatics/bts714 (PMC3570210; doi:10.1093/bioinformatics/bts714)
Supplement: Supplementary Data [file supp_bts714_supplement.pdf]

Supplement to "Data Exploration, Quality Control and Testing in Single-Cell qPCR-Based Gene Expression Experiments"

Andrew McDavid, Greg Finak, Pratip K. Chattopadyay, Maria Dominguez, Laurie Lamoreaux, Steven S. Ma, Mario Roederer and Raphael Gottardo

1 Data sets and notations

The empirical cumulative distribution of π and μ for data sets A, B, C is depicted in Supplementary Figure 1. Null wells lower the maximum value of π in experiments B and C. After filtering, this difference is no longer noted. Normal quantile-quantile plots of the robust z-transformed et for expressed genes z_{ij} are depicted in Supplementary Figure 8-10. The hypothesized normal distribution fits most genes well.

2 Derivation of combined Likelihood Ratio Statistic

Consider the composite, two-sample test:

$$H_0 : \pi_0 = \pi_1 \quad \text{and} \quad \mu_0 = \mu_1$$

versus the alternative

$$H_a : \pi_0 \neq \pi_1 \quad \text{and} \quad \mu_0 \neq \mu_1.$$

between the stimulated and un-stimulated groups. Suppose both groups share a common variance, σ^2. Omitting the gene index j for clarity, the likelihood ratio test is defined as

$$\Lambda(\mathbf{y}, \mathbf{v}) = \frac{\sup_{\boldsymbol{\theta} \in H_0} \mathrm{L}(\boldsymbol{\theta}|\mathbf{y}, \mathbf{v})}{\sup_{\boldsymbol{\theta} \in H_A} \mathrm{L}(\boldsymbol{\theta}|\mathbf{y}, \mathbf{v})} \tag{1}$$

where the likelihood is given by

$$\mathrm{L}(\boldsymbol{\theta}|\mathbf{y}, \mathbf{v}) = \prod_k \pi_k^{n_k} (1 - \pi_k)^{I - n_k} \prod_{i \in S_k} g(y_{ik}|\mu_k, \sigma^2), \tag{2}$$

$\mathbf{y}$ and $\mathbf{v}$ are the vectors of observations for the gene across the two groups, $\boldsymbol{\theta} = \{\mu_k, \sigma^2, \pi_k; k = 0, 1\}$ is the vector of unknown parameters, S_k is the set of cells expressing the gene in group k (*i.e.* $S_k = \{i : v_{ik} = 1\}$) , $n_k = \sum_i v_{ik}$ is the number of cells expressing the gene in group k, and g is the density function of the log-normal distribution with parameters μ_k and σ^2. Using the following change of variable, $et_{ik} = \log y_{ik}$, in equation (2), the likelihood function can be written as

$$\mathrm{L}(\boldsymbol{\theta}|\mathbf{et}, \mathbf{v}) = \prod_k \pi_k^{n_k} (1 - \pi_k)^{I - n_k} \prod_{i \in S_k} \mathrm{N}(et_{ik}|\mu_k, \sigma^2) \tag{3}$$

where $\mathrm{N}(\cdot|\mu, \sigma^2)$ is the density function of a normal distribution with mean μ and variance σ^2. It follows that the likelihood ratio test can be written as

$$\begin{aligned}
\Lambda(\mathbf{et}, \mathbf{v}) &= \frac{\sup_{\boldsymbol{\theta} \in H_0} \mathrm{L}(\boldsymbol{\theta}|\mathbf{et}, \mathbf{v})}{\sup_{\boldsymbol{\theta} \in H_A} \mathrm{L}(\boldsymbol{\theta}|\mathbf{et}, \mathbf{v})} \\
&= \frac{\sup_{\{\pi_0, \mu_0, \sigma^2\}} \pi_0^{n_0 + n_1} (1 - \pi_0)^{2I - n_0 - n_1} \prod_k \prod_{i \in S_k} \mathrm{N}(et_{ik}|\mu_0, \sigma^2)}{\sup_{\{\pi_0, \mu_0, \sigma^2, \pi_1, \mu_1\}} \prod_k \pi_k^{n_k} (1 - \pi_k)^{I - n_k} \prod_{i \in S_k} \mathrm{N}(et_{ik}|\mu_k, \sigma_k^2)} \\
&= \frac{\sup_{\pi_0} \pi_0^{n_0 + n_1} (1 - \pi_0)^{2I - n_0 - n_1}}{\sup_{\{\pi_0, \pi_1\}} \prod_k \pi_k^{n_k} (1 - \pi_k)^{I - n_k}} \cdot \frac{\sup_{\{\mu_0, \sigma^2\}} \prod_k \prod_{i \in S_k} \mathrm{N}(et_{ik}|\mu_0, \sigma^2)}{\sup_{\{\mu_0, \sigma^2, \mu_1\}} \prod_k \prod_{i \in S_k} \mathrm{N}(et_{ik}|\mu_k, \sigma^2)} \\
&= \Lambda_b(\mathbf{v}) \cdot \Lambda_n(\mathbf{et}^+)
\end{aligned}$$

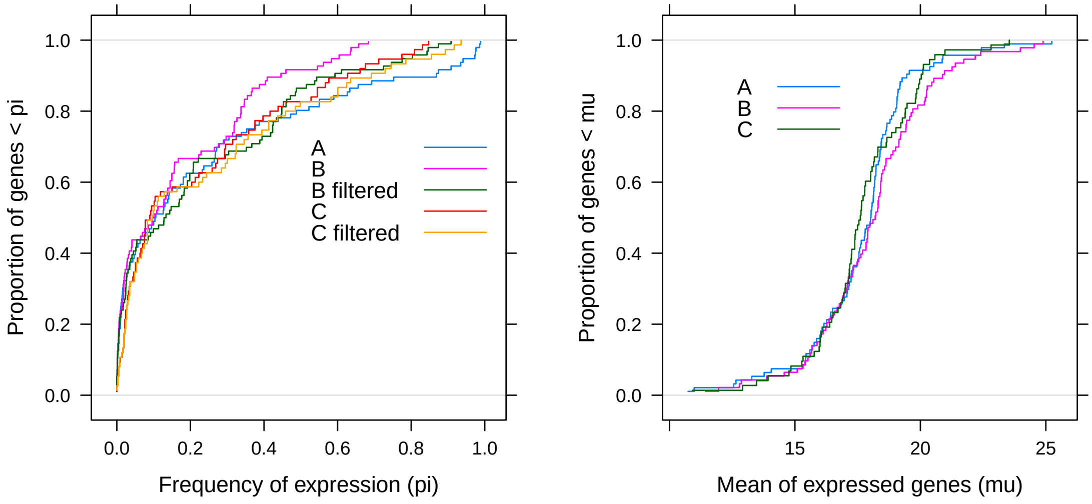

Supplementary Figure 1: Empirical cumulative distribution of π and μ for data sets A, B, C.

where Λ_b is a binomial LRT, Λ_n is a normal LRT and $\mathbf{et}^+$ is the set of positive et values. Thus our combined LRT can be computed as the product of a binomial and a normal LRT statistic, both of which can easily be derived using classical statistical theory.

3 Validity of Asymptotic Approximation

The χ_2^2 limiting distribution of Λ as $I \to \infty$ is convenient for power analysis and for quickly testing significance. In supplemental figure 2 we show the empirical cumulative distribution plot of Λ and of the limiting χ_2^2 distribution. Bivariate samples of $(\mathbf{y}, \mathbf{v})$ are simulated from hypothetical genes with $\pi = .02, .04, .08, .16, .32$ and sample size $I = 100$ in which there is no difference means and proportions between classes. The standard deviation is 1.3, which is the median empirical standard deviation across data sets. There is some inflation of the null distribution for smaller effective sample sizes ($\pi I < 8$), chiefly in the form of additional skewness that would result in the size of the test being higher than the nominal significance level. However, one is not forced to rely on the asymptotic distribution of Λ for assessing significance. When the effective sample size of the continuous component, πI is too small, *e.g.* $\pi I < 8$, the null distribution should be approximated using permutations [1].

3.1 Departures from Normality

Supplemental figure 3 depicts the same scenario as in supplemental figure 2, however now $\mathbf{v}$ is simulated from a t-distribution with 4 degrees of freedom, and scaled by 1.3, the standard deviation used in the prior simulation. This allows the assessment of the level of the test when the continuous distribution does not follow a normal distribution. As expected, since the Λ_n depends on the mean of $\mathbf{et}^+$, the central limit theorem results in this mean converging in distribution to a normal distribution, hence the test is somewhat robust to departures from normality. The null distributions are very close to the ones obtained under the normal assumption.

4 Filtering Parameter Optimization

We determine appropriate values of the continuous parameter t_z and the expression proportion parameter t_ζ by searching the grid $t_z, t_\zeta \in [3, 5, \ldots, 9]$.For each value in the grid, the weighted residual sum of squares $\overline{\text{WSS}}$ is calculated. The minimizing values vary somewhat on the data set, so we based our recommendation of $t_z = t_\zeta = 9$ by choosing values that minimize the maximum residual $\overline{\text{WSS}}$ across data sets.

Supplementary Figure 4-6 depicts the hundred-cell/single-cell concordance and $\overline{\text{WSS}}$ for data set A for $t_z = 9, \ldots, 3$ and $t_\zeta = 9, \ldots, 3$ The points depict the position of a gene after filtering at a given stringency. Since there is a natural nesting of the parameters, we consider them cumulatively and allow t_z to vary fastest. Thin lines indicate the position

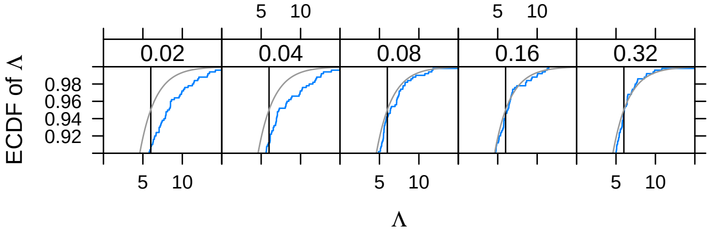

Supplementary Figure 2: The empirical cumulative distribution plot of Λ. The cumulative distribution of χ^2_2 is plotted in gray. 5% significance is indicated by a vertical line. The gene frequency π varies. The sample size $I = 100$.

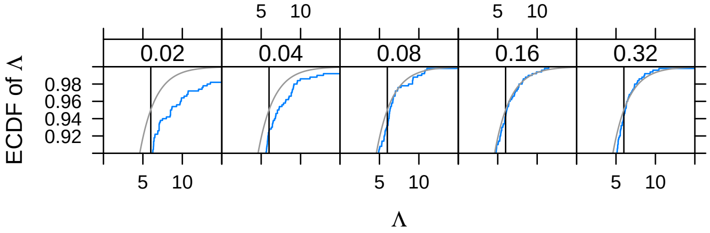

Supplementary Figure 3: The empirical cumulative distribution plot of Λ and of χ^2_2, considering departures from normality in the continuous component $\mathbf{v}$. $\mathbf{v}$ is now simulated from a t distribution with 4 degrees of freedom.

of a gene at the previous filtering stringency. Thus, for example in Supplementary Figure 4, between $(t_z, t_\zeta) = (9, 3)$ and $(t_z, t_\zeta) = (7, 3)$ one additional cell is filtered, so there is one thin, dark line, whereas at $(t_z, t_\zeta) = (5, 3)$, two genes move to the y-axis.

Supplementary Table 1 shows the $\overline{\text{WSS}}$ in all three data sets for all points in the grid described above.

4.1 Effect of Filtering on Control Genes

The flow cytometric sorting of the data sets we consider allows examination of how filtering affects the quality of the sorted cells. In data sets B and C, the cells were putatively sorted to be CD4+, and CD8- using surface markers. In data set A, the cells were sorted to be CD8+, CD4-. The flow sorting is based on protein presence, while the gene expression is based on mRNA, so it's expected that there will be differences between the two that reflect biological differences between transcription and translation.

Nonetheless, with the proviso that it would be surprising for there to be 100% concordance between translated protein and transcribed mRNA, these genes still may serve limited roles as positive and negative controls. As seen in supplemental table 2, in two out of the three data sets (A and C), the percentage of unexpected transcript decreases substantially after filtering. In B, little change is noted in either positive control or negative control genes. Since this unexpected transcript could reflect contamination or assay failure, and the filtering is agnostic to the presence or absence of any particular gene, this provides additional evidence beyond $\overline{\text{WSS}}$ that filtering may improve sample quality. The insubstantial changes in the positive control genes (CD8 for A, CD4 for B and C) likely reflects the overall rareness of filtering ($< .5\%$) in any of the data sets.

In this calculation, the reference group was taken to be all wells with detected expression in at least one gene, hence already includes step one of the filtering algorithm described in section 2.3 of the main text. If we take the reference group to be the raw measurements, then substantial increases in the expression of positive control genes are noted in B and C (comparison not shown).

5 Filtering and housekeeping genes

Scatter plots and linear fits (Supplementary Figure 7) between housekeeping genes and other frequently expressed genes suggest filtering suffices to remove technical artifacts, so normalization is unnecessary.

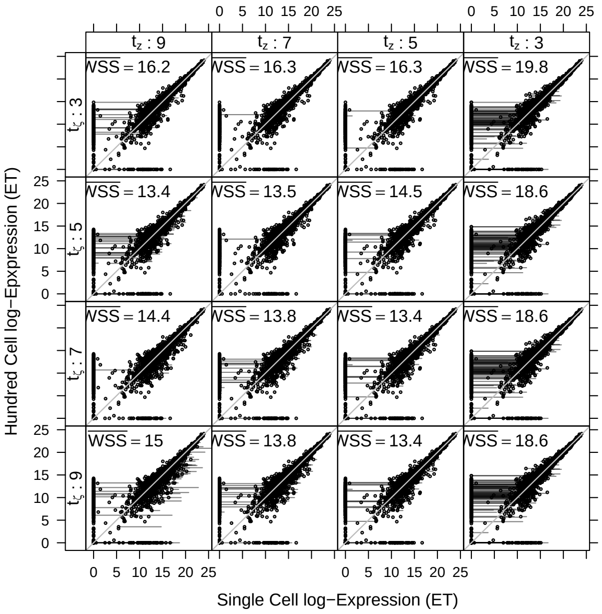

Supplementary Figure 4: Hundred cell-single cell concordance as filtering stringency increases from bottom left to upper right; data set A. Unfiltered $\overline{\mathrm{WSS}}$ is 23.2.

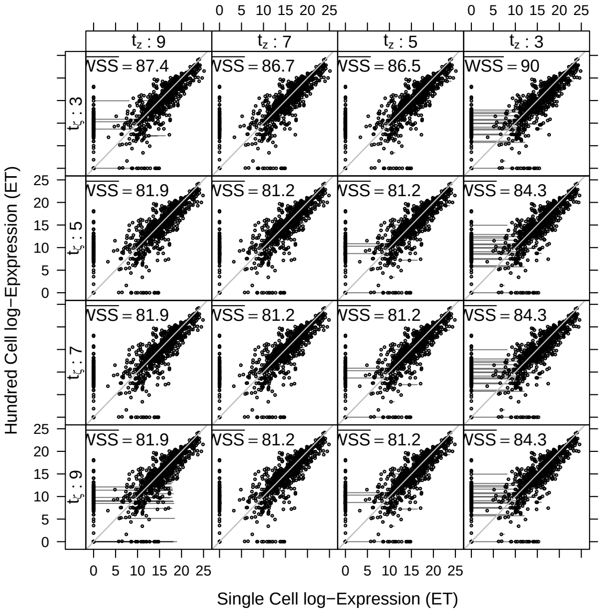

Supplementary Figure 5: Hundred cell-single cell concordance as filtering stringency increases from bottom left to upper right; data set B. Unfiltered $\overline{\mathrm{WSS}}$ is 90.1.

		t_z			
Data set	t_ζ	3	5	7	9
A	3	6.44	2.95	2.95	2.83
A	5	5.26	1.17	0.11	0.00
A	7	5.26	0.01	0.40	1.02
A	9	5.21	0.00	0.43	1.64
B	3	8.85	5.33	5.56	6.28
B	5	3.18	0.00	0.04	0.69
B	7	3.18	0.00	0.04	0.69
B	9	3.18	0.00	0.04	0.69
C	3	27.99	16.88	8.56	8.56
C	5	27.98	8.90	7.08	6.13
C	7	25.84	6.41	4.55	0.00
C	9	25.84	6.41	4.55	0.00

Supplementary Table 1: $\overline{\text{WSS}} - \min_{t_z,t_\zeta} \overline{\text{WSS}}$ values across data sets and filtering parameters. For each data set, the minimum $\overline{\text{WSS}}$ is subtracted so that cells that achieve that value contain zeroes.

Data set	PCT Filtered	CD4 Before	CD4 After	PCT Change CD4	CD8 Before	CD8 After	PCT Change CD8
A	0.301	0.013	0.011	-16.893	0.879	0.879	0.001
B	0.155	0.424	0.423	-0.028	0.002	0.002	0.155
C	0.267	0.484	0.485	0.268	0.016	0.013	-16.443

Supplementary Table 2: Effect of filtering, beyond the effect of excluding null wells, on control genes CD4 and CD8. Data set A is expected to be positive for CD8, negative for CD4. Data sets B and C are expected to be negative for CD8, positive for CD4. The percentage of cells filtered, the frequencies π before and after filtering and percentage change in π of these genes is printed.

Outlying and high-leverage points–most of which are flagged for filtering–drive most of the apparent correlation between genes, since there is very little trend in the central portion of the distribution.

References

[1] Youngchao Ge, Sandrine Dudoit, and Terence P. Speed. Resampling-based multiple testing for microarray data analysis. *TEST*, 12(1):1–77, June 2003.

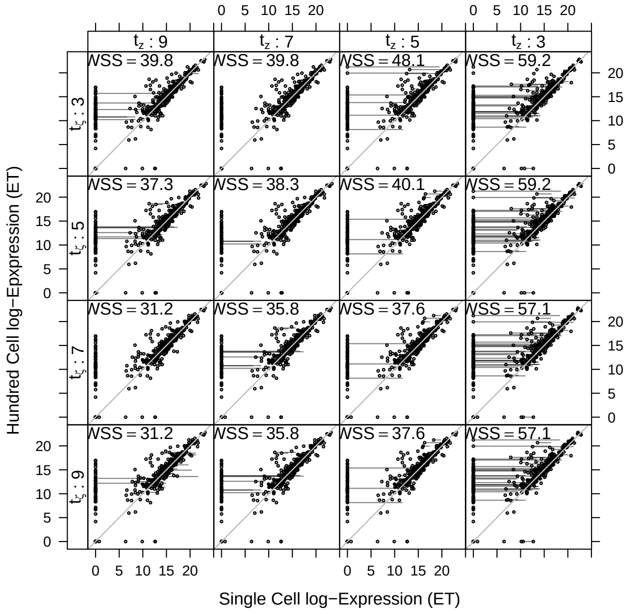

Supplementary Figure 6: Hundred cell-single cell concordance as filtering stringency increases from bottom left to upper right; data set C. Unfiltered $\overline{\mathrm{WSS}}$ is 32.

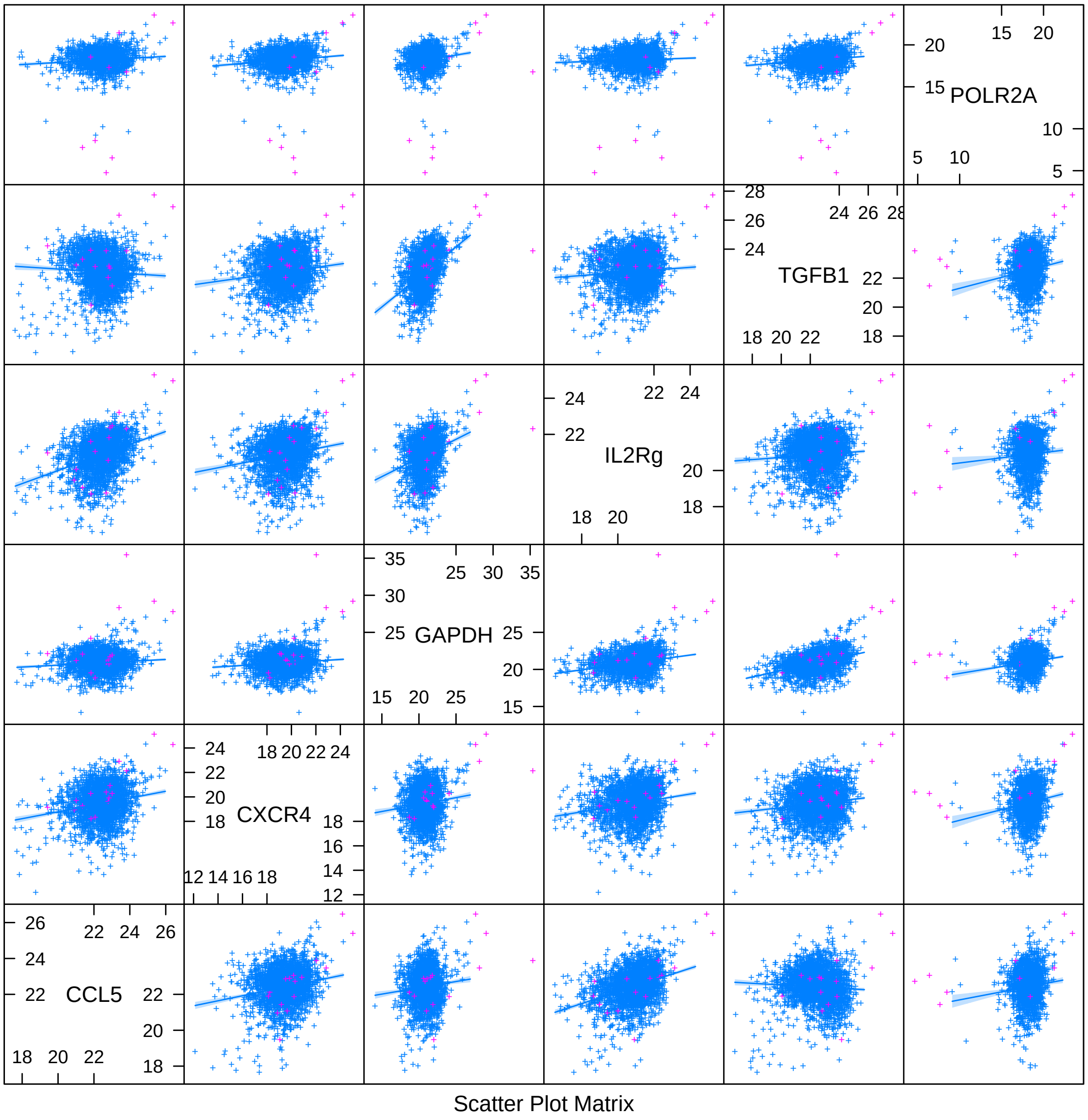

Supplementary Figure 7: Scatter plots of housekeeping genes GAPDH, POLR2A and other frequently expressed ($\pi > .95$) genes. Cells flagged for filtering are indicated in purple. A regression line of the form $et_y \sim et_x +$ intercept, and its standard error is plotted using unfiltered cells.

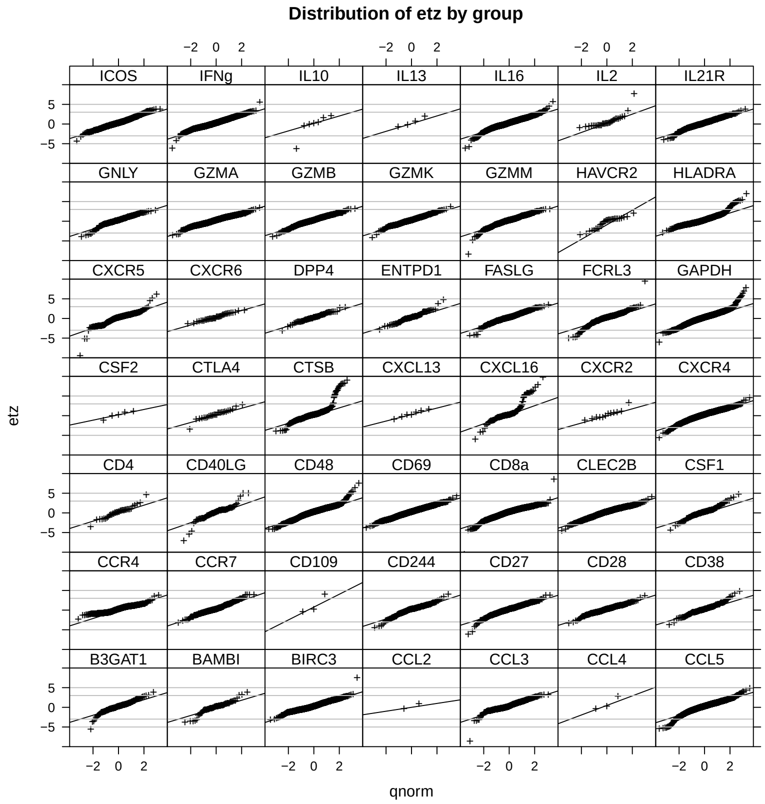

Supplementary Figure 8: Normal quantile-quantile plots of z_{ij} for 49 genes, data set A.

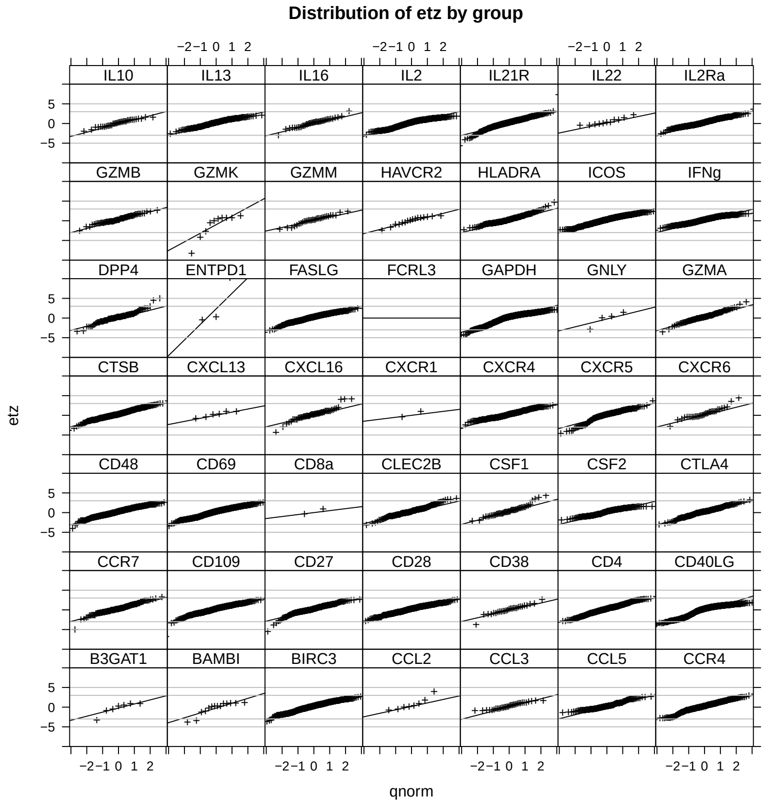

Supplementary Figure 9: Normal quantile-quantile plots of z_{ij} for 44 genes, data set B.

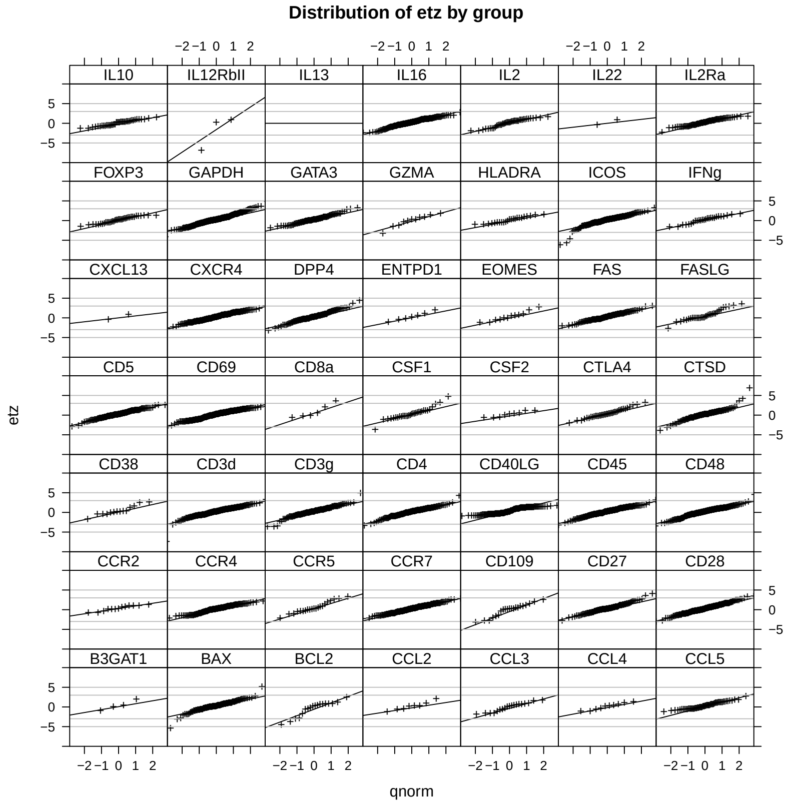

Supplementary Figure 10: Normal quantile-quantile plots of z_{ij} for 49 genes, data set C.
